# Prediction of incident chronic kidney disease in a population with normal renal function and normo-proteinuria

**DOI:** 10.1371/journal.pone.0285102

**Published:** 2023-05-03

**Authors:** Seung Min Lee, Su Hwan Kim, Hyung-Jin Yoon

**Affiliations:** 1 Department of Biomedical Engineering, Seoul National University College of Medicine, Seoul, Republic of Korea; 2 Biomedical Research Institute, Seoul National University Hospital, Seoul, Republic of Korea; 3 Medical Big Data Research Center, Seoul National University Medical Research Center, Seoul, Republic of Korea; University of KwaZulu-Natal, SOUTH AFRICA

## Abstract

Regarding the irreversible clinical course of chronic kidney disease, identifying high-risk subjects susceptible to Chronic Kidney Disease (CKD) has an important clinical implication. Previous studies have developed risk prediction models identifying high-risk individuals within a group, including those who may have experienced minor renal damage, to provide an opportunity for initiating therapies or interventions at earlier stages of CKD. To date, there were no other studies developed a prediction model with quantitative risk factors to detect the earliest stage of CKD that individuals with normal renal function in the general population may experience. We derived 11,495,668 individuals with an estimated glomerular filtration rate (eGFR) ≥90 mL/min/1.73 m^2^ and normo-proteinuria, who underwent health screening ≥2 times between 2009 and 2016 from the prospective nationwide registry cohort. The primary outcome was the incident CKD, defined by an eGFR <60 mL/min/1.73 m^2^. Sex-specific multivariate Cox regression models predicting the 8-year incident CKD risk were developed. The performance of developed models was assessed using Harrell’s C and the area under the receiver operating characteristics curve (AUROC) with 10-fold cross-validation. Both men and women, who met the definition of incident CKD, were older and had more medical treatment history in hypertension and diabetes. Harrell’s C and AUROC of the developed prediction models were 0.82 and 0.83 for men and 0.79 and 0.80 for women. This study developed sex-specific prediction equations with reasonable performance in a population with normal renal function.

## Introduction

Chronic kidney disease (CKD) is a global public health problem. The global CKD prevalence of stage 3–5 is reported to be 10.6% and its prevalence is consistently high in Europe, the USA, Canada [1], and low- and middle-income countries [2]. Although the reported prevalence and deaths from CKD have increased globally [3], disease awareness is often low among many subjects [4]. CKD is often not detected until it has already advanced due to its asymptomatic nature, and as a consequence, the medical costs of patients and healthcare systems have increased [5].

Regarding the irreversible clinical course of CKD to end-stage kidney disease and the association of CKD with all-cause and cardiovascular morbidity and mortality [6], identifying high-risk subjects susceptible to CKD has important clinical implications [7]. Many studies have reported the cost-effectiveness and advantages of early identification [8] to enable delay or prevention of CKD progression by altering modifiable risk factors [9, 10]. As the benefit from accurate prediction of risk in CKD is obvious, risk prediction models for CKD have been developed and validated in Western population [11–15]. Owing to the limited applicability and validity of these algorithms to Asian population, several studies have developed prediction models for Asians [16–20]. These models demonstrated sufficient discrimination performance (c statistics; 0.7 to 0.8), but their clinical usefulness is still questionable because of their study population, limited risk measures, limited follow-up time, and/or lack of evaluation information (diagnostic analysis) [21]. Most of these previous studies were based on individuals with an eGFR >60 mL/min/1.73 m^2^ at the baseline. However, the population with eGFR ≥90 mL/min/1.73 m^2^ and with no trace of proteinuria, which is considered as a normal kidney function, may demonstrate different characteristics and CKD progression from the rest of the population [22]. We focused exclusively on the population with normal kidney function to develop prediction models and identify related risk factors using a large representative data. The developed models may provide more accurate prediction of incident CKD while the individuals are at a risk of early stage CKD and thereby provide meaningful clinical tool facilitating early intervention and treatment to improve clinical outcomes.

Therefore, we conducted a study to develop and validate risk prediction models for the Korean population with normal renal function. The developed prediction models were based on 11,495,668 adults who had undergone two or more health screenings during 2009 and 2016 and had a baseline eGFR ≥90 mL/min/1.73 m^2^ and negative dipstick proteinuria.

## Methods

### Study population

**Fig 1:**
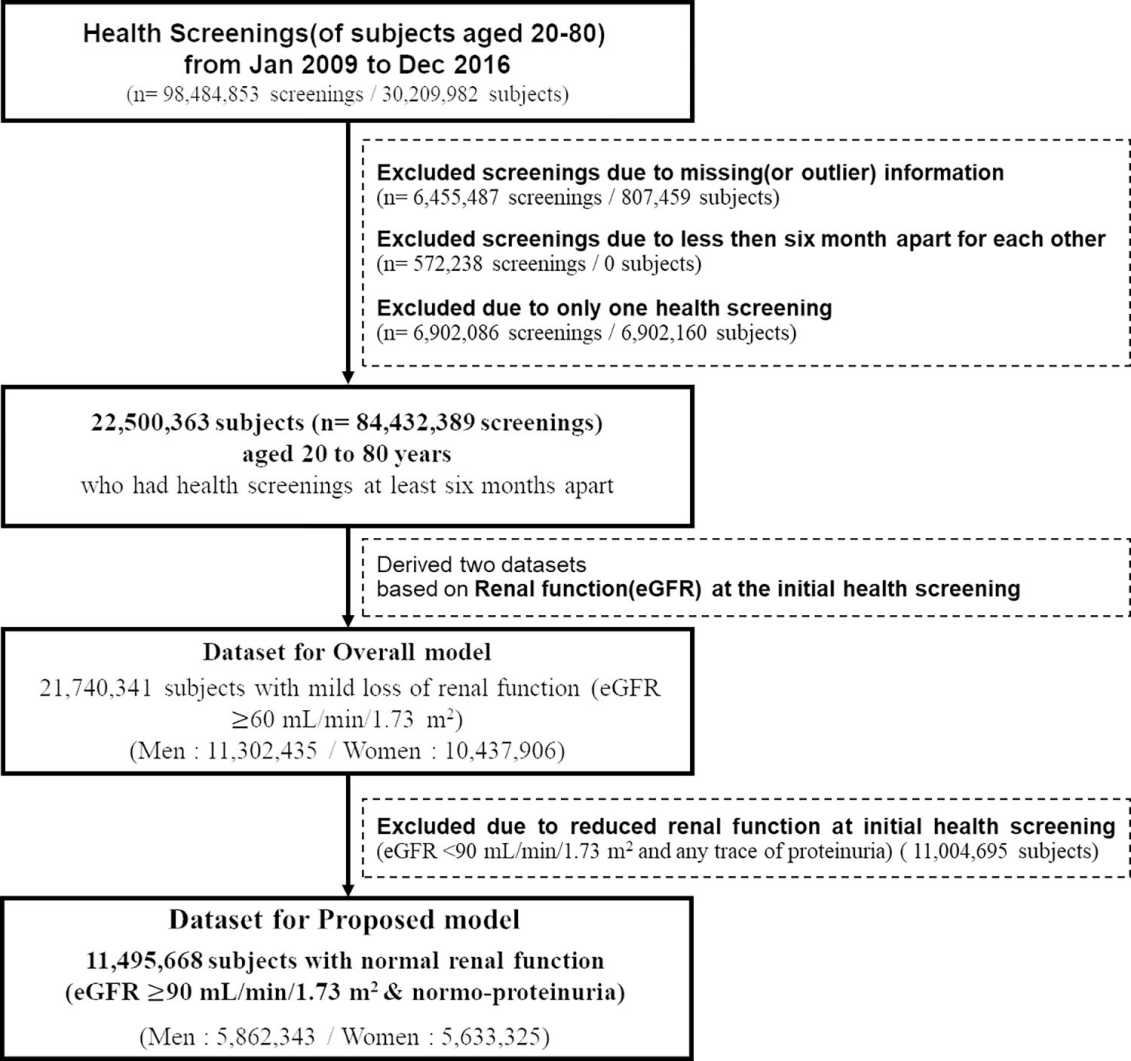
Flowchart of the subject selection.

This study was conducted using the national registry database derived from the National Health Insurance Service (NHIS) in South Korea. The NHIS collects all national health screening results for all Koreans and provides these data for the purpose of policy and academic research [23]. In Korea, all insured subjects have a legal obligation to participate in regular national health screening programs. During the program, they were asked to complete a self-reported standard questionnaire and undergo a routine health check-up at designated screening hospitals. From 2009 to 2016, 98,484,853 health screenings were performed on 30,209,982 subjects aged between 20 and 80 years. The specific information for the measurements in screening data is well-described in elsewhere [24]. The screening data of 6,561,245 screenings (807,459 subjects) were excluded due to missing or outlier values. Cutoff values for identifying outliers in this study were defined after thorough review of the distribution of the data as follows: Height (100–200[cm]), weight (25–200[kg]), waist circumference (40–125[cm]), body mass index (BMI; 13–50[kg/m^2^]), systolic blood pressure (SBP; 60–250[mmHg]), diastolic blood pressure (DBP; 40–150[mmHg]), fasting serum glucose (FSG; 1–800[mg/dL]), serum gamma-glutamyl transferase (GGT; 1–2000[U/L]), serum glutamic oxaloacetic transaminase (SGOT; 1–2900[U/L]), glutamic pyruvic transaminase (SGPT; 1–2900[U/L]), total serum cholesterol (5–740[mg/dL]), serum high-density lipoprotein cholesterol (HDL; 1–150[mg/dL]), serum low-density lipoprotein cholesterol (LDL; 1–870[mg/dL]), serum triglyceride (TG; 5–1000[mg/dL]), blood hemoglobin (10.8–17.3[g/dL]), and serum creatinine(0.2–3.7[mg/dL]). A total of 11,004,695 subjects (29,402,523 screenings), who had not completed health screening more than twice, which were performed at least six months apart, during the study period, or those with a baseline eGFR <90 mL/min/1.73 m^2^ or dipstick proteinuria (trace or higher) were excluded (The same process has been done for subjects with eGFR ≥60 mL/min/1.73 m^2^ regardless of their proteinuria status, and a total of 21,740,341 subjects were included). A total of 11,495,668 subjects (5,862,343 men and 5,633,325 women) were included in developing our proposed CKD risk prediction equations (Fig 1). The Institutional Review Board of Seoul National University Hospital (E-1505-034-670) waived the requirement for informed consent and approval because of the nature of this study, which retrospectively analyzed the national registry data.

The health screening data included a structured questionnaire, clinical measurements, and laboratory tests. Past medical history (stroke, heart diseases, hypertension, diabetes mellitus, and hyperlipidemia), family history (stroke, heart disease, hypertension, and diabetes mellitus), and lifestyle factors (smoking status, drinking habit, and physical activity) were collected using a structured questionnaire of the specified form. Height, weight, waist circumference, and blood pressure were measured. The laboratory tests for urine and blood were fasting serum sugar, liver function test, blood hemoglobin, serum lipids (total cholesterol, high-density lipoprotein cholesterol, and triglycerides), serum creatinine, and urine protein using a urine dipstick. Drinking habits were categorized using the World Health Organization (WHO) classification [25]: (1) no drinking; (2) low risk: average daily alcohol consumption <40 g/day for men and <20 g/day for women; (3) medium risk: average daily alcohol consumption between 40–59.9 g/day for men and 20–39.9 g/day for women; (4) high risk: average daily alcohol consumption ≥60 g/day for men and ≥40 g/day for women. The daily average alcohol consumption was calculated as follows: Average daily alcohol consumption (g/day) = [Frequency (drinking days in a week) × Quantity (number of drinks per day) × Volume of drink (50cc) × Alcohol by volume (0.2) × Density of alcohol (0.785)] /7 (days/week), where a standard drink is a glass of “*Soju* (distilled liquor commonly consumed in Korea).” Physical activity was defined as total metabolic equivalent task minutes per week (MET-min/week) using the International Physical Activity Questionnaire (IPAQ) and categorized into 3 levels [26]: low (<600 METs-min/week), moderate (600–2999 METs-min/week), and high (≥3000 METs-min/week). BMI was categorized by following the definition of the WHO [27]: underweight, <18.5 kg/m^2^; normal, 18.5–24.9 kg/m^2^; overweight, 25.0–29.9 kg/m^2^; and obese, ≥30.0 kg/m^2^. Incident CKD was defined as the development of low eGFR <60 mL/min/1.73 m^2^ during follow-up. The eGFR was calculated using the Chronic Kidney Disease Epidemiology Collaboration (CKD-EPI) equation based on serum creatinine level [28].

### Model development

Two sets of sex-specific multivariate Cox regression models were developed to estimate the individual risk for incident CKD: one utilizing data of all the subjects with eGFR ≥60 mL/min/1.73 m^2^ regardless of their proteinuria status (referred to as the overall model; S1 Table) and the other utilizing data of the subjects with eGFR ≥90 mL/min/1.73 m^2^ and normo-proteinuria (referred to as the proposed model; Table 2). Univariate Cox regression was performed using all available baseline variables: age, waist circumference, BMI, SBP, DBP, FSG, total serum cholesterol, HDL, LDL, TG, GGT, SGOT and SGPT, blood hemoglobin, eGFR, past medical history (diabetes mellitus, hypertension, heart disease, stroke, and hyperlipidemia), family history (stroke, heart disease, hypertension, and diabetes mellitus), smoking status (never, past, and current smokers), physical activity (low, moderate, and high activity), and alcohol intake (low, medium, and high risk). Even with the use of bi-variable selection that did not consider confounding effects, all these variables were included in the multivariable analysis with statistical significance. Our intent was to develop a model using a subset of potentially relevant factors, which is both statistically parsimonious and clinically applicable and useful. To achieve this, a stepwise selection process was used to elaborate the final multivariate Cox regression model, and risk factors with statistical significance were selected. The 10-fold cross-validation was performed to test the predicted 8-year risk of incident CKD. Discrimination was assessed using Harrell’s C and the area under the receiver operating characteristics curve (AUROC) [29, 30]. We performed diagnostic analyses by setting threshold values with the Youden index [31]. Youden’s index, sensitivity+specificity-1, with bootstrapping procedure was used to find a cutoff point that maximizes sensitivity and specificity at the same time with equal importance/weight. To assess the predictive power of the proposed model, we divided the vulnerable group (subjects with eGFR between 60–90 mL/min/1.73 m^2^ or positive dipstick proteinuria) and the less vulnerable group (subjects with eGFR ≥ 90 mL/min/1.73 m^2^ and negative dipstick proteinuria) into 10 subgroups and computed the sensitivity and specificity of the incident CKD prediction using both the proposed model and the overall model. A two-sided *p* value less than 0.05, was considered statistically significant. Analyses were performed using SAS version 9.4 (SAS Institute, Cary, NC, USA) and R 3.5.2 (http://www.R-project.org).

## Results

Of the 11,495,668 subjects (5,862,343 men and 5,633,325 women) aged 20–80 years, 187,767 subjects (71,192 men and 116,575 women) developed incident CKD during a median follow-up of 6.2 years (6.3 years for men and 6.1 years for women). The CKD incidences per 100,000 person-years were 208.2 for men and 365.1 for women. Table 1 shows the descriptive characteristics of the study subjects. Both men and women, who met the definition of incident CKD, were older and had more medical treatment history. In particular, a high proportion of CKD was observed in the medical treatment history of hypertension (26.2% in men and 26.8% in women) and diabetes (12.5% in men and 9.5% in women).

**Table 1:** Baseline general characteristics of the subjects according to sex.

Variables	Men (n = 5,862,343)		P-value	Women (n = 5,633,325)		P-value
	Control	Incident CKD		Control	Incident CKD	
	(n = 5,791,151)	(n = 71,192)		(n = 5,516,750)	(n = 116,575)	
eGFR (mL/min/1.73 m^2^)	104.3 ± 10.3	98.8 ± 8.2	<0.0001	106.2 ± 10.9	98.9 ± 7.6	<0.0001
Age (year)	40.1 ± 12.0	55.3 ± 11.2	<0.0001	42.8 ± 12.7	56.7 ± 10.7	<0.0001
Systolic Blood pressure (mm Hg)	123.2 ± 13.3	128.3 ± 15.4	<0.0001	116.7 ± 14.5	124.7 ± 16.2	<0.0001
Diastolic Blood pressure (mm Hg)	77.1 ± 9.5	79.7 ± 10.2	<0.0001	72.7 ± 9.8	76.7 ± 10.2	<0.0001
Waist circumference (cm)	82.7 ± 8.1	85.1 ± 7.6	<0.0001	74.6 ± 8.8	79.2 ± 8.7	<0.0001
Fasting serum glucose (mg/dL)	96.7 ± 23.1	106.0 ± 35.2	<0.0001	92.8 ± 18.1	98.9 ± 25.4	<0.0001
GGT (U/L)	46.4 ± 57.8	54.5 ± 73.8	<0.0001	21.0 ± 24.3	24.9 ± 28.0	<0.0001
SGPT (U/L)	29.5 ± 28.2	29.4 ± 25.1	0.1318	19.1 ± 19.4	22.5 ± 19.1	<0.0001
SGOT (U/L)	26.7 ± 20.5	28.6 ± 23.6	<0.0001	21.9 ± 15.8	24.7 ± 20.7	<0.0001
Serum total cholesterol (mg/dL)	190.0 ± 35.4	193.1 ± 37.5	<0.0001	190.2 ± 35.7	200.9 ± 38.4	<0.0001
HDL (mg/dL)	53.0 ± 12.9	51.4 ± 13.6	<0.0001	59.7 ± 13.9	55.9 ± 13.9	<0.0001
LDL (mg/dL)	109.0 ± 33.7	109.3 ± 36.6	0.0283	110.4 ± 33.2	119.1 ± 36.5	<0.0001
Serum triglyceride (mg/dL)	142.0 ± 98.0	162.6 ± 107.6	<0.0001	99.9 ± 63.2	129.0 ± 77.2	<0.0001
Blood Hemoglobin (g/dL)	15.1 ± 1.1	14.6 ± 1.2	<0.0001	13.0 ± 0.9	13.0 ± 1.0	<0.0001
Body Mass Index (kg/m^2^)			<0.0001			<0.0001
<18.5	2.6%	1.7%		7.3%	2.4%	
18.5–24.9	62.6%	57.5%		70.1%	60.2%	
25–29.9	30.8%	37.3%		19.4%	32.4%	
≥30.0	4.0%	3.5%		3.2%	5.0%	
Smoking status			<0.0001			<0.0001
Nonsmoker	30.3%	32.3%		93.2%	95.5%	
Past smoker	20.6%	28.5%		2.4%	1.3%	
Smoker	49.1%	39.2%		4.4%	3.2%	
Alcohol intake *			<0.0001			<0.0001
No drinking	28.8%	38.9%		67.7%	83.4%	
Low risk	63.4%	53.0%		28.9%	15.1%	
Medium risk	4.9%	5.0%		2.7%	1.2%	
High risk	2.9%	3.1%		0.7%	0.3%	
Physical Activity *			<0.0001			<0.0001
Low activity	35.4%	37.1%		43.8%	45.5%	
Moderate activity	53.9%	49.0%		48.4%	44.5%	
High activity	10.6%	14.0%		7.8%	10.0%	
Past medical history						
Heart Disease	1.0%	2.8%	<0.0001	0.7%	2.8%	<0.0001
Stroke	0.6%	1.2%	<0.0001	0.3%	0.9%	<0.0001
Hypertension	7.3%	26.2%	<0.0001	8.4%	26.8%	<0.0001
Diabetes mellitus	3.3%	12.5%	<0.0001	2.7%	9.5%	<0.0001
Hyperlipidemia	1.4%	3.1%	<0.0001	1.7%	4.6%	<0.0001
Family history						
Heart disease	3.2%	2.7%	<0.0001	3.6%	3.1%	<0.0001
Stroke	5.0%	6.1%	<0.0001	5.5%	6.6%	<0.0001
Hypertension	9.9%	10.1%	0.0046	13.8%	13.0%	<0.0001
Diabetes mellitus	8.8%	8.0%	<0.0001	10.6%	8.9%	<0.0001

eGFR, estimated glomerular filtration rate; GGT, serum gamma-glutamyl transferase; SGPT, serum glutamic pyruvic transaminase; SGOT, serum glutamic oxaloacetic transaminase; HDL, high-density lipoprotein cholesterol; LDL, low-density lipoprotein cholesterol.

*See the Methods for details.

Note: Percentages for categorical variables; mean±standard deviation for continuous variables; P-value was calculated by *t*-test for continuous variables and Pearson’s chi-squared test for categorical variables.

Table 2 shows adjusted hazard ratios (aHR) for CKD risk factors obtained from the proposed Cox regression models. In both men and women, the risk of incident CKD was positively associated with continuous covariates such as age, SBP, DBP, FSG, SGOT, and TG, and those under the categories of BMI (≥25 kg/m^2^), current smoker, high-intensity exercise, and medical treatment history (hypertension and diabetes). The highest risks of incident CKD occurred in both sexes with diabetes (aHR 1.38 [95% confidence interval [CI]; 1.34–1.41] for men and 1.29 [95% CI; 1.26–1.32] for women). Higher waist circumference and a history of past smoking were positively associated with a slight risk of incident CKD in men, while the medical history of treatment in heart disease was positively associated with a risk of incident CKD only in women. Those who had higher baseline eGFR, higher HDL, higher hemoglobin, underweight BMI, alcohol intake of any amount, and positive family history of heart disease, stroke, or hypertension had a decreased risk of incident CKD in both men and women. LDL and medical history of stroke were negatively associated with risk of incident CKD only in men, and a family history of diabetes mellitus was negatively associated with risk of incidence CKD only in women.

**Table 2:** Adjusted hazard ratios for incident CKD for final Cox regression models for normal renal function in men and women in the study.

Variables	Men	Women
	Hazard Ratios (95% CI)	Hazard Ratios (95% CI)
Age	1.083 (1.083–1.084)	1.069 (1.068–1.070)
Systolic Blood pressure (mmHg)	1.003 (1.002–1.003)	1.002 (1.001–1.002)
Diastolic Blood pressure (mmHg)	1.008 (1.007–1.009)	1.005 (1.004–1.006)
Waist circumference (cm)	1.010 (1.008–1.011)	0.999 (0.998–1.000)
Fasting serum glucose (mg/dL)	1.002 (1.002–1.002)	1.001 (1.001–1.001)
GGT (U/L)	1.000 (1.000–1.000)	1.000 (1.000–1.001)
SGPT (U/L)	0.999 (0.999–1.000)	1.000 (0.999–1.000)
SGOT (U/L)	1.001 (1.001–1.001)	1.001 (1.001–1.001)
Serum total cholesterol (mg/dL)	1.003 (1.002–1.004)	1.000 (1.000–1.000)
HDL (mg/dL)	0.996 (0.995–0.997)	0.999 (0.999–1.000)
LDL (mg/dL)	0.997 (0.997–0.998)	-
Serum triglyceride (mg/dL)	1.001 (1.001–1.001)	1.001 (1.001–1.001)
Hemoglobin (g/dL)	0.872 (0.866–0.878)	0.937 (0.931–0.943)
Baseline eGFR (mL/min/1.73 m^2^)	0.988 (0.987–0.989)	0.977 (0.976–0.977)
Body Mass Index (kg/m^2^)		
<18.5	0.799 (0.753–0.847)	0.868 (0.835–0.902)
25.0–29.9	1.149 (1.127–1.171)	1.111 (1.094–1.128)
≥30.0	1.101 (1.049–1.155)	1.190 (1.151–1.229)
Smoking status		
Past smoker	1.025 (1.006–1.045)	0.998 (0.949–1.050)
Smoker	1.089 (1.069–1.109)	1.130 (1.093–1.168)
Alcohol intake*		
Low risk	0.843 (0.829–0.857)	0.941 (0.925–0.957)
Medium risk	0.759 (0.732–0.787)	0.832 (0.788–0.879)
High risk	0.745 (0.712–0.779)	0.835 (0.755–0.923)
Physical Activity *		
Moderate activity	1.014 (0.998–1.03)	0.990 (0.978–1.002)
High activity	1.053 (1.028–1.077)	1.027 (1.006–1.048)
Medical history of Treatment		
Heart Disease	1.043 (0.994–1.093)	1.193 (1.151–1.236)
Stroke	0.802 (0.747–0.861)	1.019 (0.958–1.083)
Hypertension	1.379 (1.353–1.406)	1.182 (1.164–1.200)
Diabetes mellitus	1.375 (1.340–1.411)	1.294 (1.264–1.324)
Hyperlipidemia	0.992 (0.949–1.038)	0.999 (0.971–1.028)
Family history		
Heart disease	0.880 (0.839–0.923)	0.947 (0.915–0.979)
Stroke	0.917 (0.889–0.947)	0.950 (0.928–0.972)
Hypertension	0.961 (0.937–0.986)	0.964 (0.947–0.982)
Diabetes mellitus	-	0.962 (0.942–0.982)

GGT, serum gamma-glutamyl transferase; SGPT, serum glutamic pyruvic transaminase; SGOT, serum glutamic oxaloacetic transaminase; HDL, high-density lipoprotein cholesterol; LDL, low-density lipoprotein cholesterol; eGFR, estimated glomerular filtration rate.

*See the Methods for details.

Note: Covariates was selected using stepwise selection; The survival rate at the mean values of the risk factors, *S_o_*(*t*) is 0.99218 for men and 0.98547 for women.

In the discriminatory analysis performed with the 10-fold cross-validation technique, the average Harrell’s C-statistics were 0.818 for men and 0.785 for women. The average AUROCs for men and women were 0.827 and 0.801, respectively, for the proposed model. Applying a validation set of subjects with normal renal function using the overall model (eGFR ≥60), the average AUROCs for men and women were 0.815 and 0.796, respectively.

Applying the Youden index to find the maximum overall sensitivity and specificity in the proposed models resulted in a cutoff point of 0.0177 for men and 0.0346 for women. These cutoff points yielded a sensitivity of 74.7% and specificity of 75.2% in men and a sensitivity of 66.9% and specificity of 77.6% in women. The same analyses have been taken to the overall model (eGFR ≥60) to determine the cutoff points. Then, 10-fold cross-validation was applied to the subjects with normal renal function to assess the discriminating power of each model for subjects with normal renal function. The main purpose of the CKD prediction model is to allow early detection of CKD and apply appropriate preventive measures before renal damage becomes irreversible. For this reason, we compared the sensitivity and specificity of the overall and proposed models (Table 3), constructed and validated using different datasets. First, the overall model was constructed from all subjects with (eGFR ≥60 mL/min/1.73 m^2^) and the proposed model was constructed from subjects with normal renal function (eGFR ≥90 mL/min/1.73 m^2^ and negative dipstick proteinuria), which is subset of the former group of subjects. The proposed model demonstrated higher sensitivity while maintaining a sufficient level of specificity in subjects with normal renal function, demonstrating a stronger discriminating power. Moreover, the proposed model demonstrated high sensitivity for subjects with renal damage, indicating that it may serve as a screening tool for not only subjects with normal renal function but also subjects with renal damage. Schoenfeld residual plots were used to test whether the proportional hazard assumption was satisfied for each variable in both the proposed model and the overall model for men and women. No variables violated this assumption.

**Table 3:** Diagnostic characteristics of the predictions of subjects with normal renal functions.

Model	Subjects	Proposed model^1)^		Overall model^2)^	
		Sensitivity	Specificity	Sensitivity	Specificity
Men	Less vulnerable group^3)^	74.71%	75.15%	26.75%	75.15%
		(74.18–75.25)	(74.86–75.43)	(26.30–96.43)	(74.86–75.43)
	Vulnerable group^4)^	90.95%	43.53%	69.57%	78.09%
		(90.87–91.02)	(43.48–43.58)	(69.13–70.01)	(77.73–78.45)
Women	Less vulnerable group^3)^	66.90%	77.59%	39.73%	92.25%
		(66.03–67.77)	(76.92–78.26)	(39.21–40.25)	(92.12–92.39)
	Vulnerable group^4)^	92.26%	35.64%	74.63%	66.27%
		(92.20–92.32)	(35.60–35.69)	(74.47–74.80)	(66.14–66.39)

^1)^ Cox regression model developed using subjects with eGFR ≥90 mL/min/1.73 m^2^ and negative dipstick proteinuria and validated through 10-fold cross validation.

^2)^ Cox regression model developed using subjects with eGFR between 60–90 mL/min/1.73 m^2^ or positive dipstick proteinuria and validated through 10-fold cross validation.

^3)^ Subjects with eGFR ≥90 mL/min/1.73 m^2^ and negative dipstick proteinuria.

^4)^ Subjects with eGFR between 60–90 mL/min/1.73 m^2^ or positive dipstick proteinuria.

## Discussion

Using the nationwide registry data and all clinically relevant variables, we developed and validated the sex-specific equations for 8-year CKD risk prediction in the general population with normal renal function. Our risk prediction equations included most of the variables that were previously recognized as renal risk factors [32, 33]. We combined the results of stepwise selections from both the proposed and overall models into one final model in order to evaluate the significance of developing proposed model (normal renal function). The final set of predictors for incident CKD in men were age, SBP, DBP, waist circumference, FSG, GGT, SGPT, SGOT, total cholesterol, HDL, LDL, TG, blood hemoglobin, eGFR, BMI, alcohol intake, smoking status, physical activity, past medical history (heart disease, stroke, hypertension, diabetes mellitus, and hyperlipidemia), and family history (heart disease, stroke, and hypertension). For the men, only family history of diabetes mellitus was excluded. For the women, all other variables were included in the final set except LDL cholesterol. Following the general risk prediction equations [34], parameters and specifications for our equations are shown in S2 Table. Derived risk estimations from our equations for men and women demonstrated good discrimination in the AUROC and Harrell’s C calculated with 10-fold cross-validation.

In previous studies [11, 13–16, 18, 20], CKD risk prediction models were developed with subjects who might have potential kidney damage. A few Asian models have been developed that incorporate such potential risks. Prediction models for CKD stages I–V [35] were developed previously [17] but no information about the exclusion criteria of the baseline subjects was mentioned. A recent study excluded subjects with stage 3–5 CKD or dipstick proteinuria (2+ or 3+) at the initial health screening [19]. This may indicate that subjects with baseline renal damage might have been included in their study. Unlike any other study, subjects with an eGFR ≤90 mL/min/1.73 m^2^ or dipstick proteinuria (trace or higher) at the initial health check-up were excluded from our study; therefore, our prediction models may be more suitable for identifying the CKD risk factors and risks of subjects with healthy renal function. As our study design is different from other studies (included potential risk group), a direct comparison between models is not possible, but the discrimination of our models (C statistics and AUC: 0.818 and 0.827 for men and 0.785 and 0.801 for women, respectively) is fairly acceptable compared to other recently developed Asian long-term prediction models (C statistics 0.826 for men and 0.827 for women [19], and AUC 0.79 for both sexes [16]). The sensitivity for the proposed model was 74.7% (95% CI: 74.2%-75.3%) and the overall model was 26.8% (26.3%-27.2%) for men, whereas the sensitivity for the proposed model was 66.9% (66.0%-67.8%) and the overall model was 39.7% (39.2%-40.3%) for women. This shows that the selected predictors in the proposed model were reflected in many renal risk factors known to be associated with an increased risk of CKD, which may help clinicians interpret the results and discuss intervention strategies for the risk of CKD.

The association between lifestyle factors and CKD has been reported, emphasizing that lifestyle factors may play a significant role in developing CKD [36, 37]. All lifestyle factors (smoking, drinking, and physical activity) were selected in our final prediction model, but our study has found a reverse association between incident CKD and alcohol intake, contrary to the general knowledge. Our prediction model did not account for all possible phenomena like hidden reverse epidemiology since our primary goal was to construct a prediction model with variables increasing the predictive power rather than an inference model. Smoking had adverse effects on CKD in our study, and the same result was seen in a case-control study by Yacoub et al. [38], who reported that current smokers have an increased risk of CKD incidence compared to nonsmokers. Physical activity(exercising for 30 minutes, 5 times per week) is recommended as a lifestyle intervention for subjects with CKD by the Kidney Disease Improving Global Outcomes [39]. Our finding showed that the high activity group (≥3000 METs-min/week) was positively associated with the risk of incident CKD. A similar result was reported in a cross-sectional study by Wang et al. [40] indicating that the high-intensity exercise group was associated with decreased eGFR (≤90 mL/min/1.73 m^2^). These results imply the importance of the proper intensity and time of exercise for high-risk subjects with incident CKD, and the possible differential association between physical activity and CKD needs to be confirmed in future studies. Our study showed a protective effect of alcohol intake on the risk of incident CKD, similar to previous observations elsewhere [41–44]. This negative association between alcohol intake and incident CKD should be interpreted cautiously because higher alcohol consumption is reported to be associated with an increased risk of overall mortality [45]. Our findings suggest that future studies on these complex relationships of lifestyle should be conducted to clarify the association with CKD. Most of all, it is advisable to adopt a healthy lifestyle to reduce the risk of not only CKD but also other chronic health conditions.

The strengths of the current study include its study design and methodology. This study developed prediction models to estimate the risk of CKD in subjects with healthy renal function (eGFR >90 mL/min/1.73 m^2^) and no trace of proteinuria, based on large cohorts. We used the national registry database of a large number of Koreans aged 20–80 (~22 million). According to the Korean Statistical Information Service [46], the estimated general population aged 20–79 years in 2010 was 35 million. By using the data of about two-thirds of the general population, we were able to develop CKD risk prediction equations with less selection bias. Moreover, we developed and validated our prediction equations using 10-fold cross-validation to reduce bias and variance. All available variables were tested statistically, and only validated variables were selected for our final prediction equations to provide enough information for interpretation and understanding of the estimated risk of CKD. Lastly, we provided the diagnostic characteristics of different cutoff points to improve the detection of high-risk for CKD; the evaluation of CKD risk with cutoff points may help physicians to decide whether close monitoring or additional testing is necessary.

This study has some limitations. First, the definition of outcome was based on eGFR (<60 mL/min/1.73 m^2^) using the CKD-EPI equation instead of measured GFR. The measurement of GFR is not practical in most studies with large sample size, such as ours. Second, there can be misclassification of variables reported by subjects in a self-reported standard questionnaire. Although a standard questionnaire was designed to be clear and easy to understand, the possibility of mistakes could not be excluded. Finally, risk prediction equations were developed in ethnic Korean subjects; therefore, caution may be necessary when planning implementation to groups whose lifestyles are different from the Korean general population.

In summary, we developed and validated sex-specific risk prediction equations for incident CKD in a general population with a baseline eGFR ≥90 mL/min/1.73 m^2^ and negative dipstick proteinuria. To the best of our knowledge, this is the first study to develop and validate CKD risk prediction equations in subjects with normal renal function. The discriminative ability of the final prediction equations showed that the performance of the model was fairly acceptable, and the selected risk factors in the equations would provide an additional interpretation and understanding of subjects with normal renal function at a high risk of CKD. The clinical utility of these equations must be validated in other populations.

## Supporting information

S1 Table Adjusted hazard ratios for incident CKD for final Cox regression models for all male and female included in the study (overall model). (DOCX)

S2 Table Specific examples to estimate CKD risk using the proposed risk prediction equations. (DOCX)

S3 Table Baseline general characteristics of the subjects by gender. (DOCX)
