# Supplementary material for: Prediction of incident chronic kidney disease in a population with normal renal function and normo-proteinuria
Source: PLoS One. 2023 May 3;18(5):e0285102. doi: 10.1371/journal.pone.0285102 (PMC10155979; doi:10.1371/journal.pone.0285102)
Supplement: S1 Table — (DOCX) [file pone.0285102.s001.docx]

**S1 Table. Adjusted hazard ratios for incident CKD for final Cox regression models for all male and female included in the study (overall model)**

| **Variables** | **Men** | **Women** |
| --- | --- | --- |
|  | Hazard Ratios (95% CI) | Hazard Ratios (95% CI) |
| Age | 1.060 (1.060-1.060) | 1.053 (1.052-1.053) |
| Systolic Blood pressure (mmHg) | 1.003 (1.003-1.004) | 1.002 (1.002-1.002) |
| Diastolic Blood pressure (mmHg) | 1.004 (1.004-1.005) | 1.003 (1.002-1.003) |
| Waist circumference (cm) | 1.006 (1.005-1.006) | 1.002 (1.001-1.002) |
| Fasting serum glucose (mg/dL) | 1.001 (1.001-1.001) | 1.000 (1.000-1.001) |
| GGT (U/L) | 1.000 (1.000-1.000) | 1.001 (1.000-1.001) |
| SGPT (U/L) | 0.999 (0.999-0.999) | 0.999 (0.999-1.000) |
| SGOT (U/L) | 1.000 (1.000-1.000) | 1.001 (1.001-1.001) |
| Serum total cholesterol (mg/dL) | 1.001 (1.000-1.001) | 1.000 (1.000-1.000) |
| HDL (mg/dL) | 0.995 (0.995-0.996) | 0.998 (0.997-0.998) |
| LDL (mg/dL) | 0.999 (0.999-1.000) | - |
| Serum triglyceride (mg/dL) | 1.001 (1.000-1.001) | 1.000 (1.000-1.000) |
| Hemoglobin (g/dL) | 0.952 (0.949-0.954) | 0.952 (0.949-0.954) |
| Baseline eGFR (mL/min/1.73 m2) | 0.958 (0.958-0.958) | 0.958 (0.958-0.958) |
| Body Mass Index (kg/m2) |  |  |
| <18.5 | 0.811 (0.792-0.831) | 0.836 (0.819-0.853) |
| 25.0-29.9 | 1.107 (1.099-1.115) | 1.092 (1.085-1.100) |
| ≥30.0 | 1.124 (1.104-1.144) | 1.165 (1.149-1.182) |
| Smoking status |  |  |
| Past smoker | 1.034 (1.027-1.042) | 1.004 (0.980-1.029) |
| Smoker | 1.069 (1.062-1.077) | 1.096 (1.079-1.114) |
| Alcohol intake* |  |  |
| Low risk | 0.893 (0.888-0.899) | 0.919 (0.911-0.927) |
| Medium risk | 0.794 (0.782-0.806) | 0.811 (0.787-0.835) |
| High risk | 0.779 (0.764-0.794) | 0.773 (0.731-0.817) |
| Physical Activity * |  |  |
| Moderate activity | 1.015 (1.010-1.021) | 1.015 (1.010-1.021) |
| High activity | 1.054 (1.045-1.064) | 1.054 (1.045-1.064) |
| Medical history of Treatment |  |  |
| Heart Disease | 1.112 (1.097-1.128) | 1.171 (1.155-1.187) |
| Stroke | 1.014 (0.993-1.035) | 1.057 (1.032-1.082) |
| Hypertension | 1.307 (1.298-1.316) | 1.186 (1.179-1.194) |
| Diabetes mellitus | 1.347 (1.335-1.360) | 1.315 (1.302-1.328) |
| Hyperlipidemia | 1.030 (1.015-1.046) | 1.042 (1.030-1.055) |
| Family history |  |  |
| Heart disease | 0.964 (0.948-0.981) | 0.984 (0.969-0.999) |
| Stroke | 0.990 (0.979-1.001) | 0.997 (0.986-1.007) |
| Hypertension | 1.020 (1.011-1.029) | 1.002 (0.994-1.010) |
| Diabetes mellitus | - | 0.994 (0.984-1.003) |
| Proteinuria |  |  |
| Trace or >1+ | 1.484 (1.470-1.499) | 1.271 (1.257-1.285) |

GGT, serum gamma-glutamyl transferase; SGPT, serum glutamic pyruvic transaminase; SGOT, serum glutamic oxaloacetic transaminase; HDL, high-density lipoprotein cholesterol; LDL, low-density lipoprotein cholesterol; eGFR, estimated glomerular filtration rate.
