# Supplementary material for: Prediction of incident chronic kidney disease in a population with normal renal function and normo-proteinuria
Source: PLoS One. 2023 May 3;18(5):e0285102. doi: 10.1371/journal.pone.0285102 (PMC10155979; doi:10.1371/journal.pone.0285102)
Supplement: S2 Table — (DOCX) [file pone.0285102.s002.docx]

**S2 Table. Specific examples to estimate CKD risk using the proposed risk prediction equations**

| **Variables** | **Men** | | | **Women** | | |
| --- | --- | --- | --- | --- | --- | --- |
|  | An individual with agaed 40 years with SBP/DBP 124/70 mmHg, Waist circumference 80 cm, fasting serum glucose 93 mg/dL, GGT 20 U/L, SGPT 21 U/L, SGOP 32 U/L, total cholesterol 143 mg/dL, HDL-C 54 mg/dL, LDL-C 76 mg/dL, triglyceride 63 mg/dL, Hemoglobin 5.2 g/dL, eGFR 91.31 mL/min/1.73 m^2^, body mass index 26.1 kg/m^2^, smoker, low-risk alcohol intakes, moderate physical activity, no treatment history, and the family history of hypertension. | | | | | |
|  | **Coefficient (**$\boldsymbol{\beta}$**)** | **Individual value(X)** | $\boldsymbol{\beta}\left( \boldsymbol{X}\mathbf{-}\boldsymbol{M} \right)$ | **Coefficient (**$\boldsymbol{\beta}$**)** | **Individual value(X)** | $\boldsymbol{\beta}\left( \boldsymbol{X}\mathbf{-}\boldsymbol{M} \right)$ |
| Age | 0.08009 | 40 | -0.02243 | 0.06691 | 40 | -0.20341 |
| Systolic Blood pressure (mmHg) | 0.00267 | 124 | 0.00203 | 0.00179 | 124 | 0.01282 |
| Diastolic Blood pressure (mmHg) | 0.00815 | 70 | -0.05835 | 0.00524 | 70 | -0.01478 |
| Waist circumference (cm) | 0.00965 | 80 | -0.02586 | -0.00074 | 80 | -0.00391 |
| Fasting serum glucose (mg/dL) | 0.00218 | 93 | -0.00831 | 0.00099 | 93 | 0.00009 |
| GGT (U/L) | -0.00014 | 20 | 0.00367 | 0.00033 | 20 | -0.00035 |
| SGPT (U/L) | -0.00091 | 21 | 0.00771 | -0.00022 | 21 | -0.00041 |
| SGOT (U/L) | 0.00092 | 32 | 0.00516 | 0.00078 | 32 | 0.00783 |
| Serum total cholesterol (mg/dL) | 0.00298 | 143 | -0.14012 | -0.00032 | 143 | 0.01523 |
| HDL (mg/dL) | -0.00371 | 54 | -0.00393 | -0.00069 | 54 | 0.00392 |
| LDL (mg/dL) | -0.00255 | 76 | 0.08425 | - | 76 | 0.00000 |
| Serum triglyceride (mg/dL) | 0.00080 | 63 | -0.06312 | 0.00115 | 63 | -0.04316 |
| Hemoglobin (g/dL) | -0.13732 | 5.2 | 1.35261 | -0.06493 | 5.2 | 0.50450 |
| Baseline eGFR (mL/min/1.73 m^2^) | -0.01204 | 91.31 | 0.46848 | -0.02376 | 91.31 | 0.34903 |
| Body Mass Index (kg/m^2^) |  |  |  |  |  |  |
| <18.5 | -0.22489 | 0 | 0.00584 | -0.14178 | 0 | 0.01019 |
| 25.0-29.9 | 0.13861 | 1 | 0.09555 | 0.10514 | 1 | 0.08450 |
| ≥30.0 | 0.09589 | 0 | -0.00397 | 0.17359 | 0 | -0.00563 |
| Smoking status |  |  |  |  |  |  |
| Past smoker | 0.02493 | 0 | -0.00515 | -0.00186 | 0 | 0.00004 |
| Smoker | 0.08535 | 1 | 0.04352 | 0.12209 | 1 | 0.11672 |
| Alcohol intake* |  |  |  |  |  |  |
| Low risk | -0.17109 | 1 | -0.06284 | -0.06121 | 1 | -0.04370 |
| Medium risk | -0.27544 | 0 | 0.01341 | -0.18335 | 0 | 0.00482 |
| High risk | -0.29431 | 0 | 0.00853 | -0.18041 | 0 | 0.00132 |
| Physical Activity * |  |  |  |  |  |  |
| Moderate activity | 0.01375 | 1 | 0.00634 | -0.01031 | 1 | -0.00533 |
| High activity | 0.05122 | 0 | -0.00548 | 0.02644 | 0 | -0.00206 |
| Medical history of Treatment |  |  |  |  |  |  |
| Heart Disease | 0.04184 | 0 | -0.00044 | 0.17641 | 0 | -0.00131 |
| Stroke | -0.22084 | 0 | 0.00145 | 0.01850 | 0 | -0.00005 |
| Hypertension | 0.32153 | 0 | -0.02430 | 0.16725 | 0 | -0.01475 |
| Diabetes mellitus | 0.31852 | 0 | -0.01100 | 0.25769 | 0 | -0.00720 |
| Hyperlipidemia | -0.00757 | 0 | 0.00010 | -0.00104 | 0 | 0.00002 |
| Family history |  |  |  |  |  |  |
| Heart disease | -0.12770 | 0 | 0.00407 | -0.05463 | 0 | 0.00195 |
| Stroke | -0.08619 | 0 | 0.00430 | -0.05175 | 0 | 0.00287 |
| Hypertension | -0.03933 | 1 | -0.03543 | -0.03649 | 1 | -0.03148 |
| Diabetes mellitus | - | - |  | -0.03913 | 0 | 0.00412 |
|  |  |  |  |  |  |  |
| Total sum, f(x,M) |  |  | 1.32286 |  |  | 0.74244 |
| So(t) |  |  | 0.99218 |  |  | 0.98547 |
| Estimated 8-year risk (%) * |  |  | 2.904 |  |  | 3.028 |

GGT, serum gamma-glutamyl transferase; SGPT, serum glutamic pyruvic transaminase; SGOT, serum glutamic oxaloacetic transaminase; HDL, high-density lipoprotein cholesterol; LDL, low-density lipoprotein cholesterol; eGFR, estimated glomerular filtration rate.

*** The estimated 8-year risk of the first CKD event is calculated based on the formula below:**

Predicted CKD risk = 1 - $S_{o}{(t)}^{e^{(f[x,.M])}}$

, where f(x,M) =$\beta_{1}\left( x_{1}-M_{1} \right)+\ldots+\beta_{n}\left( x_{n}-M_{n} \right)$; $S_{o}\left( t \right)$ is the survival rate at the mean values of the risk factors; $\beta_{1}$.. $\beta_{n}$ are the coefficient, $x_{1}$ … $x_{n}$ are the an individual’s values; and $M_{1}$ … $M_{n}$ are the mean value of the risk factors in our dataset (S3 table)
