# Supplementary material for: Prediction of incident chronic kidney disease in a population with normal renal function and normo-proteinuria
Source: PLoS One. 2023 May 3;18(5):e0285102. doi: 10.1371/journal.pone.0285102 (PMC10155979; doi:10.1371/journal.pone.0285102)
Supplement: S3 Table — (DOCX) [file pone.0285102.s003.docx]

**S3 Table. Baseline general characteristics of the subjects by gender**

| **Variables** | **Men (n= 5,862,343)** | **Women (n= 5,633,325)** |
| --- | --- | --- |
| eGFR (mL/min/1.73 m^2^) | 104.22 ± 10.25 | 106.00 ± 10.89 |
| Age (year) | 40.28 ± 12.09 | 43.04 ± 12.83 |
| Systolic Blood pressure (mm Hg) | 123.24 ± 13.33 | 116.84 ± 14.59 |
| Diastolic Blood pressure (mm Hg) | 77.16 ± 9.51 | 72.82 ± 9.79 |
| Waist circumference (cm) | 82.68 ± 8.09 | 74.73 ± 8.81 |
| Fasting serum glucose (mg/dL) | 96.81 ± 23.31 | 92.91 ± 18.28 |
| GGT (U/L) | 46.53 ± 58.00 | 21.07 ± 24.41 |
| SGPT (U/L) | 29.50 ± 28.16 | 19.15 ± 19.46 |
| SGOT (U/L) | 26.73 ± 20.58 | 21.91 ± 15.88 |
| Serum total cholesterol (mg/dL) | 190.02 ± 35.41 | 190.44 ± 35.74 |
| HDL (mg/dL) | 52.94 ± 12.91 | 59.66 ± 13.93 |
| LDL (mg/dL) | 109.04 ± 33.75 | 110.56 ± 33.31 |
| Serum triglyceride (mg/dL) | 142.29 ± 98.18 | 100.53 ± 63.69 |
| Blood Hemoglobin (g/dL) | 15.05 ± 1.05 | 12.97 ± 0.93 |
| Body Mass Index (kg/m^2^) |  |  |
| <18.5 | 2.59% | 7.19% |
| 18.5-24.9 | 62.51% | 69.94% |
| 25-29.9 | 30.87% | 19.63% |
| ≥30.0 | 4.03% | 3.24% |
| Smoking status |  |  |
| Nonsmoker | 30.32% | 93.26% |
| Past smoker | 20.67% | 2.35% |
| Smoker | 49.01% | 4.41% |
| Alcohol intake ^*^ |  |  |
| No drinking | 28.96% | 68.03% |
| Low risk | 63.27% | 28.61% |
| Medium risk | 4.87% | 2.63% |
| High risk | 2.90% | 0.73% |
| Physical Activity ^*^ |  |  |
| Low activity | 35.44% | 43.86% |
| Moderate activity | 53.87% | 48.34% |
| High activity | 10.69% | 7.80% |
| Past medical history |  |  |
| Heart Disease | 1.06% | 0.74% |
| Stroke | 0.66% | 0.29% |
| Hypertension | 7.56% | 8.82% |
| Diabetes mellitus | 3.45% | 2.79% |
| Hyperlipidemia | 1.37% | 1.72% |
| Family history |  |  |
| Heart disease | 3.19% | 3.57% |
| Stroke | 4.98% | 5.55% |
| Hypertension | 9.91% | 13.74% |
| Diabetes mellitus | 8.74% | 10.52% |
| Incident CKD events | 1.21% | 2.07% |

eGFR, estimated glomerular filtration rate; GGT, serum gamma-glutamyl transferase; SGPT, serum glutamic pyruvic transaminase; SGOT, serum glutamic oxaloacetic transaminase; HDL, high-density lipoprotein cholesterol; LDL, low-density lipoprotein cholesterol.

^*^See the Methods for details.

Note: Percentages for categorical variables; mean±standard deviation for continuous variables
